# Ensemble All Time-Scale Decomposition Method and Its Application in Bevel Gear Fault Diagnosis

**DOI:** 10.3390/s25010023

**Published:** 2024-12-24

**Authors:** Zhengyang Cheng, Yu Yang, Chengcheng Duan, Xin Kang, Jianxin Cui

**Affiliations:** College of Mechanical and Vehicle Engineering, Hunan University, Changsha 410082, China; amoreczy@hnu.edu.cn (Z.C.); duanchengcheng@hnu.edu.cn (C.D.); kx15939269509@hnu.edu.cn (X.K.); ccccjxdyx@hnu.edu.cn (J.C.)

**Keywords:** ensemble all time-scale decomposition, noise-assisted technique, all time-scale decomposition, zero-crossing point, gear fault diagnosis

## Abstract

All time-scale decomposition (ATD) is a non-parametric adaptive signal decomposition method, which relies on zero-crossing points and extreme points to jointly construct the baseline, achieving the suppression of modal mixing caused by the proximity of component frequencies. However, ATD is unable to solve mode mixing induced by noise. To improve this defect, a new noise-assisted signal decomposition method named ensemble all time-scale decomposition (EATD) is proposed in this paper. EATD introduces the noise-assisted technique of complementary ensemble empirical mode decomposition based on ATD, adding complementary noises to mask the noise interference in the signal. EATD not only overcomes mode mixing caused by noise but also preserves the capability of ATD to suppress mode mixing caused by the proximity of component frequencies. Simulation signals and bevel gear fault signals are utilized to validate EATD, and the results indicate that EATD can successfully overcome mode mixing induced by noise and can be effectively applied for gear fault diagnosis.

## 1. Introduction

With the widespread application of industrial automation and mechanical equipment, the research of equipment fault warning and diagnosis technology is becoming increasingly important [1,2]. Gears, as critical components in mechanical transmission systems, can suffer faults that not only affect equipment performance but also potentially lead to significant economic losses and safety hazards. Therefore, developing efficient and accurate gear fault diagnosis methods has become a key task for researchers.

Current fault diagnosis methods mainly include signal processing methods and pattern recognition methods. Pattern recognition methods rely on training classification models to intelligently classify vibration signals with high accuracy. Researchers have conducted extensive studies on pattern recognition methods, such as various machine learning algorithms and deep learning algorithms [3,4,5]. Signal processing methods quickly locate fault sources by directly analyzing the vibration signals of mechanical equipment. Compared to pattern recognition methods, signal processing methods are influenced by subjective factors but do not require large amounts of data to train models [6,7]. Therefore, both types of methods have their own advantages. This paper primarily conducts an in-depth study of signal processing methods.

In recent years, with the advancement of signal processing and analysis technologies, various time-frequency analysis methods have been proposed to enhance the capability to extract fault features [8,9,10]. Traditional adaptive signal decomposition methods, due to their capability to adaptively decompose non-stationary and nonlinear vibration signals, continue to play an important role in the field of fault diagnosis.

As the most typical adaptive signal decomposition method, empirical mode decomposition (EMD) [11,12] sifts signals by fitting mean curves and can adaptively decompose complex signals into multiple intrinsic mode functions with accurate instantaneous frequency features. EMD can perform signal decomposition without the need to set parameters, providing an effective method for fault feature extraction. However, the main drawback of EMD is mode mixing, which affects the accurate extraction of fault features [13,14].

As another traditional adaptive signal decomposition method, local mean decomposition (LMD) [15,16] relies on the envelope characteristics of signals to adaptively decompose complex signals into several product functions. The core processes of LMD, including sliding average processing, demodulation, and sifting, allow LMD to avoid the envelope overshoot and undershoot that exist in EMD, but LMD cannot solve mode mixing.

Local characteristic-scale decomposition (LCD) is an adaptive signal decomposition method proposed by combining the commonalities of EMD and LMD [17,18]. LCD assumes that a signal is composed of multiple intrinsic scale components (ISCs) whose instantaneous frequency has physical significance. By constructing the baseline based on the definition of ISC, LCD effectively extracts local characteristic information from the signals. Thus, LCD possesses higher frequency resolution than EMD and LMD. However, LCD still suffers from mode mixing.

Unlike the adaptive signal decomposition methods of the sift type, such as EMD, LMD, and LCD, the variational mode decomposition (VMD) [19] method calculates the center frequency and bandwidth constraints by solving a variational problem in the frequency domain, which provides greater precision in decomposition capability. Although VMD suppresses mode mixing better than adaptive signal decomposition methods of the sift type, it lacks adaptivity, and the decomposition capability is controlled by the number of predicted modes and the penalty factor. Improvements for VMD primarily focused on optimizing parameters and cannot fully resolve the issue of mode mixing [20,21].

The commonality among the above methods is the definition of mono-component signals that possess instantaneous frequency with physical significance. Although VMD fundamentally differs from signal decomposition methods of the sift type in principle, its concept also revolves around the definition of mono-component signals. These methods define mono-component signals under different conditions and then design the decomposition principles based on these conditions. The differences in the conditions of mono-component signals cause variations in decomposition capabilities. EMD and LCD actually utilize only the extreme points to define the mono-component signals, which leads to the omission of valuable characteristic information at other time scales.

Based on this idea, we recently proposed a new adaptive signal decomposition method named all time-scale decomposition (ATD) [22]. ATD defines all time-scale components (ATC) using zero-crossing points and extreme points and constructs the baseline to adaptively decompose the signal. Through the combined effect of zero-crossing points and extreme points, ATD can extract features from different time scales, enabling it to overcome mode mixing caused by proximity of component frequencies. However, the baseline of ATD captures more local feature information while increasing the sensitivity to the local waveform of the signal, resulting in ATD’s inability to achieve the ideal decomposition of noisy signals.

Aiming at this issue, a noise-assisted technique is a suitable solution. The noise-assisted technique was first proposed in ensemble empirical mode decomposition (EEMD) [23,24] to overcome the mode mixing of EMD. By utilizing the properties of normal distribution of Gaussian white noise, the noise-assisted technique adds Gaussian white noise to the signal multiple times, which can mask the interference from noise components after ensemble averaging. When decomposing signals with severe mode mixing, EEMD requires increasing the ensemble number, resulting in an increase in computation time. Another problem of EEMD is the pseudo-components because the component modes of each decomposition result are not guaranteed to be aligned. Complementary ensemble empirical mode decomposition (CEEMD) [25,26], as an improved method of EEMD, modifies the noise addition step by adding pairs of opposite Gaussian white noise, which significantly reduces the ensemble number and the number of pseudo-components. CEEMD not only saves computational resources but also enhances the accuracy of the decomposition results, indicating that its noise-assisted algorithm has more advantages than that of EEMD.

Therefore, combining the ATD method and the noise-assisted algorithm of CEEMD, this paper proposes a new noise-assisted signal decomposition method named ensemble all time-scale decomposition (EATD). First, EATD adds pairs of opposite Gaussian white noise multiple times to the signal. Subsequently, EATD utilizes the original ATD method to decompose multiple sets of signals added with Gaussian white noise. Finally, the decomposition results are acquired by ensemble averaging all mode components. EATD has the following three advantages:

1. EATD defines all time-scale components (ATCs), constructing the baseline jointly using zero-crossing points and extreme points. The constructed baseline allows EATD to achieve extremely high frequency resolution and overcome mode mixing caused by the proximity of component frequencies.
2. The noise-assisted algorithm of EATD significantly suppresses mode mixing induced by noise interference, while maintaining extremely high frequency resolution.
3. EATD can effectively reduce noise interference in the vibration signals of gears and accurately extract the components containing fault features from the signals.

As a novel noise-assisted signal decomposition method, EATD can accurately extract fault features from gear fault signals and can be effectively applied in gear fault diagnosis. The subsequent content of the paper is organized as follows: Section 2 describes the theory of EATD, Section 3 presents simulation signal analysis, Section 4 reports bevel gear fault experiments, and Section 5 concludes the paper.

## 2. Ensemble All Time-Scale Decomposition

In order to solve the issue of mode mixing in adaptive signal decomposition methods for the sift type, such as EMD, we recently proposed an all time-scale decomposition (ATD) method. Unlike EMD and similar methods, the construction of ATD’s baseline utilizes not only extreme points but also zero-crossing points, allowing ATD to capture more local feature information of the signal. This advantage enables ATD to effectively suppress mode mixing caused by the proximity of component frequencies, but ATD cannot solve mode mixing caused by noisy components. For this reason, this paper introduces the noise-assisted algorithm of CEEMD based on the ATD method and proposes a new noise-assisted signal decomposition method named ensemble all time-scale decomposition (EATD).

### 2.1. The Principle of All Time-Scale Decomposition

The ATD method treats any complex signal as consisting of multiple all time-scale components (ATCs), whose instantaneous frequencies have physical significance. The definition of ATC is detailed in reference [22]. The steps to decompose a signal X(t)(0<t≤T) using ATD are as follows:

1. The number of components i and the sift number j is initialized to 1, and the input signal $g_{i}=X(t)$.
2. All extreme points $\left(\tau_{n},y_{n}\right)$ are searched out from the input signal, where $\tau_{n}$ denotes time, and $y_{n}$ denotes the extremum (maximum or minimum). n=1,2,…,m, and m denotes the number of extrema.
3. The extreme datum sequence is represented as follows:
  (1) $$L_{n}=\left[y_{n-1}+\frac{y_{n+1}-y_{n-1}}{\tau_{n+1}-\tau_{n-1}}(\tau_{n}-\tau_{n-1})+y_{n}\right]/2,\;n=2,\ldots ,m-1$$
  Due to the finite length of the signal, the endpoints on both sides of the signal are treated as $L_{1}$ and $L_{m}$. The endpoint effect generated during decomposition is eliminated by endpoint extension.

4. The zero-crossing sequence is represented as follows:
  (2) $$R_{n}=c_{n}\;(L_{n}+L_{n+1}),\;n=2,\ldots ,m-2$$
  where $c_{n}$ is the compensation factor, as detailed in reference [22].
5. The extreme datum sequence and the zero-crossing point sequence are combined as the baseline sequence $\left(L_{1},L_{2},R_{2},L_{3},R_{3},\ldots ,R_{m-2},L_{m-1},L_{m}\right)$, and the baseline $Y_{i,j}(t)$ is generated by fitting the baseline sequence with a cubic spline interpolation function. The ATC candidate $h_{i,j}(t)$ is sifted by the following:
  (3) $$h_{i,j}(t)=g_{i}(t)-Y_{i,j}(t)$$
6. If $h_{i,j}(t)$ fulfills all conditions of ATC, $h_{i,j}(t)$ is ${\mathrm{ATC}}_{i}$. Otherwise, $h_{i,j}(t)$ becomes the input signal, and j=j+1. Steps 2 to 6 are repeated until all conditions of ATC are satisfied.
7. The residual signal $g_{i+1}$ is acquired by sifting out all known ATC from the signal X(t).
  (4) $$g_{i+1}(t)=X(t)-\sum_{u=1}^{i}{\mathrm{ATC}}_{u}$$
8. The termination condition for ATD is defined as follows:
  (5) $$\gamma =\frac{\sum_{t=0}^{T}{\left|g_{i+1}(t)\right|}^{2}}{\sum_{t=0}^{T}{\left|X(t)\right|}^{2}}\leq \gamma_{0}$$
  where $\gamma_{0}$ is the termination threshold and is typically set to 0.1. If the termination condition is satisfied, the decomposition terminates. Otherwise, $g_{i+1}(t)$ becomes the input signal, and i=i+1. Steps 2 to 8 are repeated until $\gamma \leq \gamma_{0}$. Finally, the decomposition results include i ATC components and one useless residue $g_{i+1}(t)$.

The signal X(t) can be represented as follows:

(6) $$X(t)=\sum_{u=1}^{i}{\mathrm{ATC}}_{u}+g_{i+1}(t)$$

In Step 6, since it is difficult to satisfy all the conditions of ATC when decomposing real vibration signals, it can lead to an increase in the number of iterations and is prone to generate pseudo-components. In order to solve this problem, the standard deviation (SD) is utilized instead of the conditions of ATC. The SD is represented as follows:

(7) $$\mathrm{SD}=\sum_{t=0}^{T}\left[\frac{{\left|h_{i,j}(t)-h_{i,j-1}(t)\right|}^{2}}{h_{i,j-1}^{2}(t)}\right]$$

The termination condition of the loop in Step 6 is that SD is less than the threshold. A threshold that is too large will reduce the integrity of the components, while a threshold that is too small will increase the number of iterations. In order to speed up the computation while ensuring that the components conform to the definition of ATC, the SD threshold used in this paper is set to 0.3.

### 2.2. The Principle of Ensemble All Time-Scale Decomposition

While the baseline of ATD captures more information about the local features of the signal, it also leads to greater sensitivity to the local waveform of the signal. As a result, ATD cannot realize the ideal decomposition for noisy signals. Aiming at this issue, the noise-assisted technique is considered to improve the ATD method.

The noise-assisted technique refers to the approach of adding Gaussian white noise to the signal multiple times and averaging the ensemble decomposition results. Due to the normal distribution of Gaussian white noise, the more the ensemble number, the more effectively the anomalous interference in the signal can be eliminated. The noise-assisted technique was first proposed in EEMD, but the noise-assisted technique of CEEMD is more mature and has advantages in controlling the number of pseudo-components and reducing the ensemble number. Therefore, the noise-assisted algorithm of CEEMD is introduced to improve the ATD method, and the ensemble all time-scale decomposition (EATD) method is proposed in this paper.

The steps to decompose a signal X(t)(0<t≤T) using EATD are as follows:

1. The N/2 pair of Gaussian white noises, which are opposite to each other, is added to the signal X(t).
  (8) $$\left\{\begin{array}{l} X_{i}^{+}(t)=X(t)+\mu w_{i}(t)\\ X_{i}^{-}(t)=X(t)-\mu w_{i}(t) \end{array}\right.$$
  where $w_{i}(t)$ is Gaussian white noise, and i=1,…,N/2. N is the ensemble number, and μ=λ∗std[X(t)] is the noise coefficient. std[X(t)] is the standard deviation of the signal X(t), and λ is generally set to 0.2 by default [25].
2. The components ${\mathrm{ATC}}_{i,j}^{+}$ and ${\mathrm{ATC}}_{i,j}^{-}$ are generated using ATD for $X_{i}^{+}(t)$ and $X_{i}^{-}(t)$, respectively:
  (9) $$\left\{\begin{array}{l} X_{i}^{+}(t)=\sum_{j=1}^{v}{\mathrm{ATC}}_{i,j}^{+}+r_{i}^{+}(t)\\ X_{i}^{-}(t)=\sum_{j=1}^{v}{\mathrm{ATC}}_{i,j}^{-}+r_{i}^{-}(t) \end{array}\right.$$
  where j=1,…,v, and v is the number of components.
3. Step (2) is repeated from i=1 to i=N/2.
4. The j-th component ${\mathrm{ATC}}_{j}$ is generated by performing ensemble averaging on all ATCs of order j:
  (10) $${\mathrm{ATC}}_{j}=\frac{1}{N}\sum_{i=1}^{N/2}\left({\mathrm{ATC}}_{i,j}^{+}+{\mathrm{ATC}}_{i,j}^{-}\right)$$

The signal X(t) can be represented as follows:

(11) $$X(t)=\sum_{j=1}^{v}{\mathrm{ATC}}_{j}+r(t)$$

where $r(t)=\frac{1}{N}\sum_{i}^{N/2}\left[r_{i}^{+}(t)+r_{i}^{-}(t)\right]$.

In general, the advantages of ATD are adaptability, extremely high frequency resolution, and the ability to extract fault features. Compared to widely used adaptive signal processing methods such as EMD, ATD can overcome mode mixing caused by proximity of component frequencies. However, the drawback of ATD is its inability to effectively decompose noisy signals.

As an improved method of ATD, EATD solves the issue that ATD cannot achieve the ideal decomposition of noisy signals, and the noise-assisted algorithm can significantly reduce noise interference. EATD retains the advantage of extremely high frequency resolution while also exhibiting noise robustness, further enhancing its ability to suppress mode mixing.

## 3. Simulation Signal Analysis

In this section, a noisy simulation signal is constructed for the purpose of verifying the feasibility and effectiveness of the EATD method. Since EATD belongs to the noise-assisted signal decomposition methods, two methods, CEEMD and VMD, are selected to compare with the EATD method in their capability to suppress mode mixing. The original ATD has been compared with EMD in reference [22], and the decomposition ability is superior to EMD. Therefore, the comparison between the two original methods, EMD and ATD, is not included in this paper.

**Figure 1:**
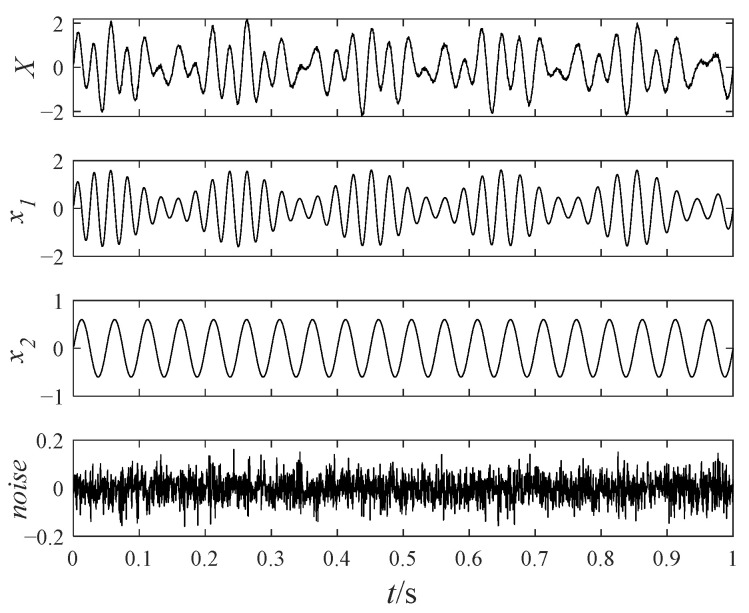
The time-domain waveforms of the noisy simulation signal.

The noisy simulation signal shown in Equation (12) contains an AM–FM signal, a sinusoidal signal, and a Gaussian white noise with a noise amplitude of 0.1. The time-domain waveforms of the noisy simulation signal are displayed in Figure 1.

(12) $$\left\{\begin{array}{l} X(t)=x_{1}(t)+x_{2}(t)+noise\\ x_{1}(t)=(1+0.6\sin(10\pi t)\sin(120\pi t-15\pi t^{2})\\ x_{2}(t)=0.6\sin(60\pi t) \end{array}\right.$$

**Figure 2:**
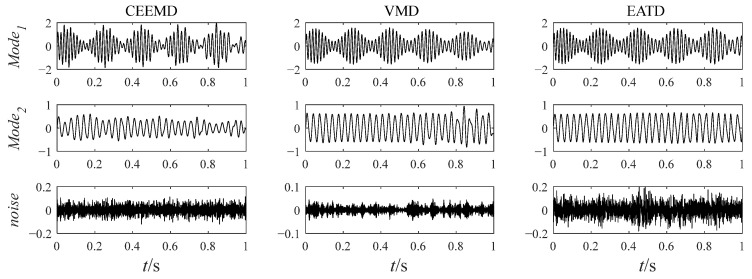
The decomposition results of CEEMD, VMD, and EATD.

The noisy simulation signal is decomposed by the three methods CEEMD, VMD, and EATD, and the results are displayed in Figure 2. To make the comparison fair, the endpoints on both sides of the signal are extended to inhibit the end effect. In addition, the parameters for the four methods are recorded in Table 1.

In Figure 2, Mode1, Mode2, and noise correspond sequentially to the two components and noise of the noisy simulation signal. Due to the excessive number of components, this paper only displays relevant components. Analysis of Figure 2 reveals that Mode1 and Mode2 of CEEMD produce obvious mode mixing. Although CEEMD can eliminate noise interference through a noise-assisted algorithm, it does not possess the capability to decompose signals that contain components with close frequencies. Mode2 of VMD exhibits mode mixing on the right side. Due to the frequency modulation characteristic of signal $x_{1}(t)$, the frequency of $x_{1}(t)$ increasingly approaches the frequency of $x_{2}(t)$ over time. This indicates that VMD’s capability to suppress mode mixing weakens as the frequencies of the components become closer. There is no mode mixing in the decomposition results of EATD, and it can be clearly observed that Mode1 and Mode2 of EATD closely match with the original components $x_{1}(t)$ and $x_{2}(t)$, which proves that the proposed EATD method is feasible.

To exclude the influence of subjective factors on the comparison conclusion, two evaluation indexes are introduced to quantify the decomposition performance, including relevant error energy ε, correlation coefficient ρ, and index of orthogonality (IO). Smaller relevant error energy and larger correlation coefficients indicate more accurate decomposition results. Smaller IO indicates better decomposition orthogonality.

Table 1 displays the parameters and evaluation indexes for CEEMD, VMD, and EATD, where $\varepsilon_{i}$ and $\rho_{i}$ represent the relevant error energy and correlation coefficient between the i-th component and the original component, respectively. All indexes of EATD possess optimal values, which proves the correctness of the conclusions derived from observing the time-domain waveforms of the decomposition results.

This simulation signal analysis demonstrates that EATD is effective for decomposing noisy signals and has advantages over CEEMD and VMD in suppressing mode mixing.

## 4. Bevel Gear Fault Experiment

When the parts of mechanical equipment malfunction, their vibration signals contain fault feature information, and different parts produce different fault features. Thus, extracting fault features can not only determine whether mechanical equipment has malfunctioned but also identify the specific location of the fault. For gear fault diagnosis, a common and reliable method is to utilize a signal decomposition method to extract the mode component containing fault features, and then assess the fault condition based on the fault feature frequency in the frequency domain. Therefore, this section will validate the effectiveness of the EATD method in decomposing real-world vibration signals.

**Figure 3:**
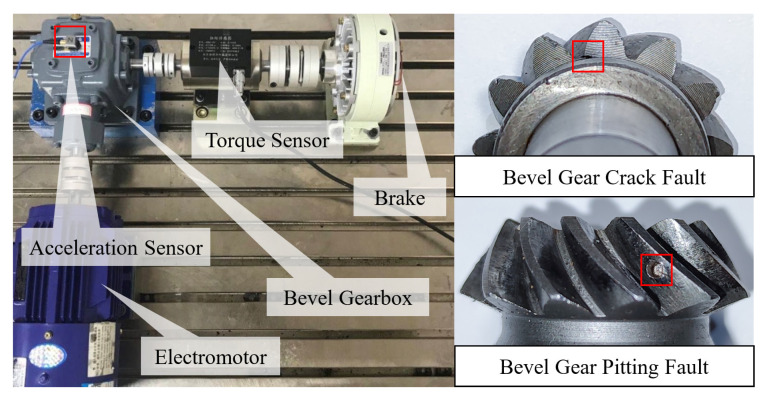
The test platform for bevel gear faults and faulty bevel gears.

For this purpose, a test platform designed for bevel gears is employed to produce bevel gear fault signals, which comprises a bevel gearbox, an electromotor, a brake, a torque sensor, and a three-axis acceleration sensor. The bevel gearbox consists of two bevel gears with 12 teeth on the driver bevel gear and 24 teeth on the driven bevel gear. Figure 3 displays the test platform and the fault type of bevel gears. The three-axis acceleration sensor of type PCB 356A25 is placed at the top center of the bevel gearbox. The type of data acquisition system is LMS SCM09.

Considering the necessity to fully validate the capability of EATD to decompose gear fault signals, two experiments with different fault types and operating conditions are planned. Experiment 1 is the signal analysis of a bevel gear with a crack fault, and Experiment 2 is the signal analysis of a bevel gear with a pitting fault. The details of the two experiments are described in Table 2.

### 4.1. Experiment 1

**Figure 4:**
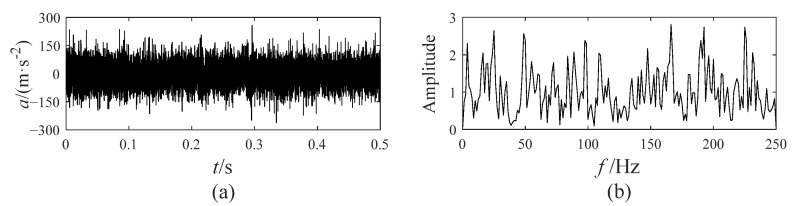
The time-domain waveform and envelope spectrum of the crack fault signal: (**a**) time-domain waveform; (**b**) envelope spectrum.

The vibration signal of the bevel gear with a crack fault is collected by the acceleration sensor. Its time-domain waveform and envelope spectrum are displayed in Figure 4.

**Figure 5:**
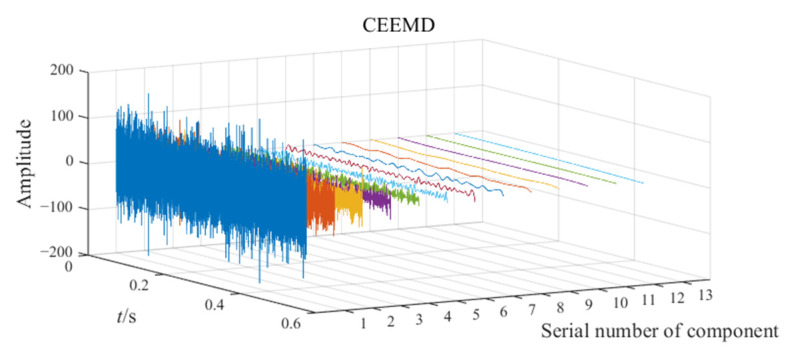
The decomposition result of CEEMD in Experiment 1.

**Figure 6:**
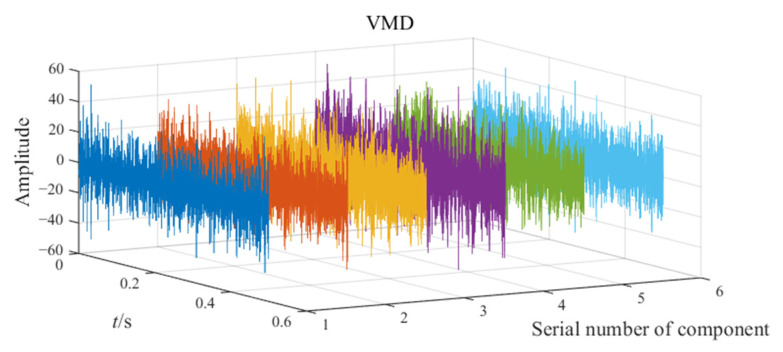
The decomposition result of VMD in Experiment 1.

**Figure 7:**
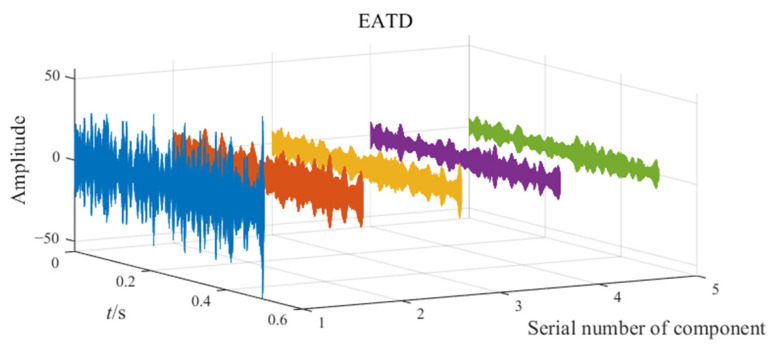
The decomposition result of EATD in Experiment 1.

In Figure 4b, a large amount of noise frequencies interfere with the diagnosis of the gear fault, but a signal decomposition method can achieve the extraction of components that contain the fault frequency from the fault signal. Besides utilizing EATD to decompose the fault signals, CEEMD and VMD are also utilized to compare with EATD. This comparison allows for an evaluation of the strengths and weaknesses of the three methods in terms of extracting fault features. The decomposition results of CEEMD, VMD, and EATD are sequentially displayed in Figure 5, Figure 6 and Figure 7. The ensemble number and the noise coefficient for CEEMD and EATD are set to 200 and 0.2, respectively. The optimal parameters for VMD are set as follows: alpha = 5000, and K = 6.

**Figure 8:**
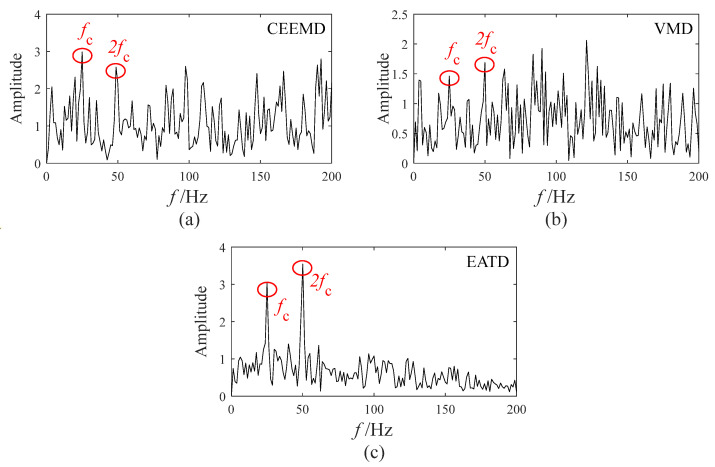
The optimal envelope spectrum in Experiment 1: (**a**) CEEMD; (**b**) VMD; (**c**) EATD.

Both impact and modulation characteristics appear in certain components of the three methods, indicating a possible fault. However, the fault feature cannot be extracted in the time-domain waveforms. Thus, an envelope spectrum analysis is required to demodulate all components of the three methods. After completing the envelope spectrum analysis, an optimal envelope spectrum is selected from the component envelope spectrum of each method according to the degree of fault feature prominence. The optimal envelope spectrums are displayed in Figure 8.

The observation from Figure 8 indicates that the fault feature frequency and its double are present in the optimal envelope spectra of CEEMD, VMD, and EATD. However, the optimal envelope spectra of CEEMD and VMD are significantly affected by numerous interferences of noise frequencies, which severely impact the gear fault diagnosis. In contrast, the optimal envelope spectrum of EATD causes an easy identification of the fault feature frequencies and their double, indicating the occurrence of gear faults. Therefore, the above experimental analysis reveals that EATD is successful in extracting fault features from gear signals. Compared to CEEMD and VMD, EATD demonstrates superior capability in the decomposition of real-world vibration signals.

### 4.2. Experiment 2

**Figure 9:**
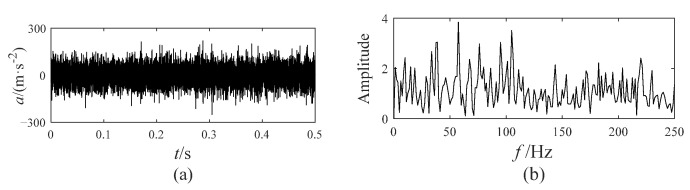
The time-domain waveform and envelope spectrum of pitting fault signal: (**a**) time-domain waveform; (**b**) envelope spectrum.

The vibration signals of the bevel gear with pitting faults are utilized to further validate the capability of EATD to extract fault features from gear signals, and its time-domain waveform and envelope spectrum are displayed in Figure 9.

**Figure 10:**
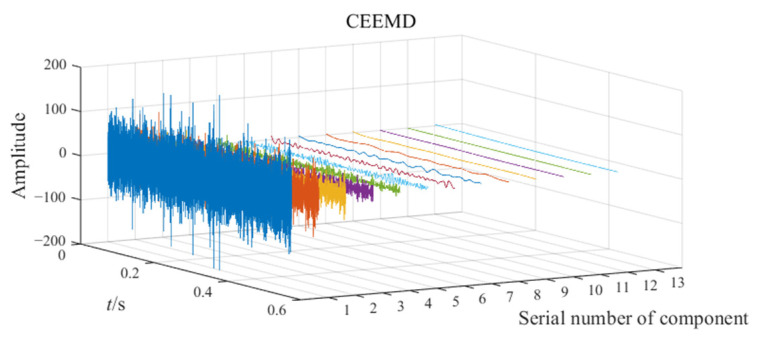
The decomposition result of CEEMD in Experiment 2.

**Figure 11:**
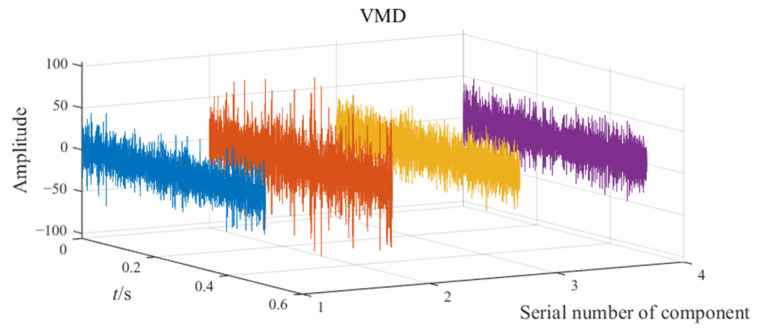
The decomposition result of VMD in Experiment 2.

**Figure 12:**
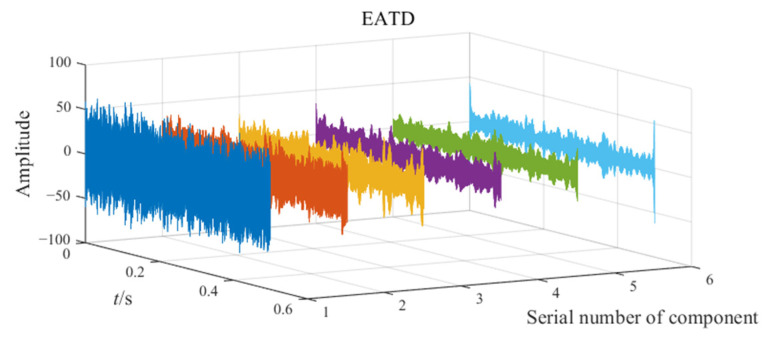
The decomposition result of EATD in Experiment 2.

In view of the difficulty in accurately locating the fault frequency in the envelope spectrum of the gear fault signal, it is essential to decompose the gear fault signal through a signal decomposition method. Therefore, the gear fault signal is decomposed using the three methods: CEEMD, VMD, and EATD, and the results are sequentially displayed in Figure 10, Figure 11 and Figure 12. The ensemble number and the noise coefficient for CEEMD and EATD are set to 200 and 0.2, respectively. The optimal parameters for VMD are set as follows: alpha = 3000, and K = 4.

Similar to Experiment 1, the faults cannot be identified from the time-domain waveforms of the components, so envelope spectrum analysis is utilized to further search for components that contain the fault feature. Once the envelope spectrums of all the components are acquired, the optimal envelope spectrum is picked from the envelope spectrum of each method.

**Figure 13:**
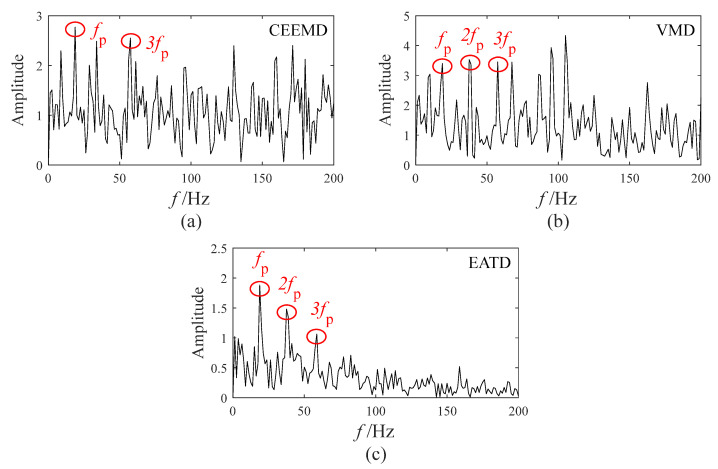
The optimal envelope spectrum in Experiment 2: (**a**) CEEMD; (**b**) VMD; (**c**) EATD.

Figure 13 displays the optimal envelope spectrum of CEEMD, VMD, and EATD. In the optimal envelope spectrums of the three methods, the fault feature frequency and its multiples appear. However, there are many noise frequencies with high amplitudes surrounding the fault feature frequency and its multiples in the optimal envelope spectrums of CEEMD and VMD, which add interference factors to identify the fault feature frequency. As a result, CEEMD and VMD cannot overcome the mode mixing present in the gear vibration signals and fail to extract gear fault features. In the optimal envelope spectrum of EATD, there is almost no interference from noise frequencies, and the fault feature frequency and its multiples can be pinpointed.

The above two experiments indicate that EATD can correctly extract the mode components containing fault features from the fault signals of a bevel gearbox. Compared with CEEMD and VMD, the decomposition performance of EATD is superior, and EATD can successfully overcome the mode mixing in real-world vibration signals.

## 5. Conclusions

Based on the ATD method, this paper introduces the complementary noise-assisted technique of CEEMD and proposes an ensemble all time-scale decomposition (EATD) method. First, EATD adds pairs of opposite Gaussian white noise multiple times to the signal. Subsequently, EATD utilizes the original ATD method to decompose multiple sets of signals added with Gaussian white noise. Finally, the decomposition results are acquired by ensemble averaging all mode components. EATD effectively utilizes complementary noises to mask noise interference, overcoming mode mixing induced by noise while retaining the capability of ATD to suppress mode mixing caused by the proximity of component frequencies. EATD is applied to analyze the simulation signals and gear fault signals and is compared with CEEMD and VMD. The results demonstrate that EATD exhibits stronger mode mixing suppression capability and decomposition performance. EATD successfully overcomes the mode mixing induced by noise and extracts gear fault characteristics, making it a valuable tool for gear fault diagnosis. However, there is still space for improvement in EATD, such as optimizing parameters and reducing pseudo-components, which are worth further study.

## Figures and Tables

**Table 1:** Parameters and evaluation indexes.

Method	Parameter	$\varepsilon_{1}$	$\varepsilon_{2}$	$\rho_{1}$	$\rho_{2}$	IO
CEEMD	N=100 , λ=0.2	0.0659	0.2099	0.9672	0.9390	0.1284
VMD	alpha=1500 , K=3	0.0273	0.0697	0.9834	0.9655	0.0905
EATD	N=100 , λ=0.2	**0.0185**	**0.0547**	**0.9907**	**0.9729**	**0.0827**

**Table 2:** The operating conditions and parameters of the two experiments.

Experiment	Faulty Gear	Fault Type	Motor Speed	Load	Sampling Frequency	Fault Feature Frequency
1	Driver Bevel Gear	Crack	1500 RPM	0 N	20,480 Hz	$f_{c}=25\;\mathrm{Hz}$
2	Driver Bevel Gear	Pitting	1200 RPM	4 N	20,480 Hz	$f_{p}=20\;\mathrm{Hz}$

## Data Availability

The data are not publicly available due to the privacy policy of the funding agency.
